# Selective MicroRNA Dysregulation in IPF‐Derived Mesenchymal Stromal Cells Suggests Restricted Regulatory Remodeling

**DOI:** 10.1096/fj.202602423RR

**Published:** 2026-09-22

**Authors:** Mariangela Di Vincenzo, Federico Mei, Paride Cavina, Francesca Gonnelli, Martina Bonifazi, Monia Orciani

**Affiliations:** ^1^ Department of Clinical and Molecular Sciences—Histology Università Politecnica Delle Marche Ancona Italy; ^2^ Department of Biomedical Sciences and Public Health Università Politecnica Delle Marche Ancona Italy; ^3^ Interstitial Lung Diseases, Pleural Diseases and Bronchiectasis Unit AOU Delle Marche Ancona Italy

## Abstract

The schematic workflow illustrates next‐generation sequencing of lung‐derived mesenchymal stromal cells (MSCs) from IPF patients and controls. Rather than broad epigenetic reprogramming, IPF‐MSCs exhibit a remarkably restricted down‐regulation of only three critical microRNAs: hsa‐miR‐373‐3p, hsa‐miR‐486‐5p, and hsa‐miR‐449a. This selective dysregulation leads to the targeted upregulation of key profibrotic genes (*SMAD4*, *BCL2*, *TGF‐β*, *CXCL12*, *VEGF*), driving TGF‐β cascade activation, altered autophagy, and profibrotic and proangiogenic signaling.

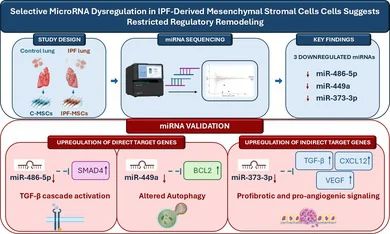

Dear Editor,

Idiopathic pulmonary fibrosis (IPF) is a progressive interstitial lung disease characterized by aberrant extracellular matrix deposition and irreversible tissue remodeling. Increasing evidence indicates that mesenchymal stromal cells (MSCs) actively contribute to fibrogenesis through altered secretory and regulatory programs [1]. MicroRNAs (miRNAs) are critical modulators of fibrotic signaling and MSC behavior [2], yet the extent of miRNA dysregulation in IPF‐derived MSCs remains poorly defined.

To investigate this issue, we performed next‐generation sequencing of MSCs isolated from lung tissue of IPF patients (*n* = 5) and nonfibrotic controls (*n* = 5). MSCs were isolated and characterized according to ISCT criteria, as previously described [1]. Consistent with these criteria, the isolated cells were plastic‐adherent, exhibited the characteristic MSC immunophenotype (CD73+, CD90+, CD105+, HLA‐DR−, CD14−, CD19−, CD34−, and CD45−), and retained the capacity to differentiate into osteoblasts, chondrocytes, and adipocytes (Figure 1). Total RNA was extracted from 3 × 10^6^ cells and libraries were sequenced on an AVITI platform (Element Biosciences). Reads were analyzed using CLC Genomics Server 25.0 with the QIAseq miRNA Quantification workflow aligned to miRBase v22. Differential expression analysis was performed using EdgeR.

**FIGURE 1:**
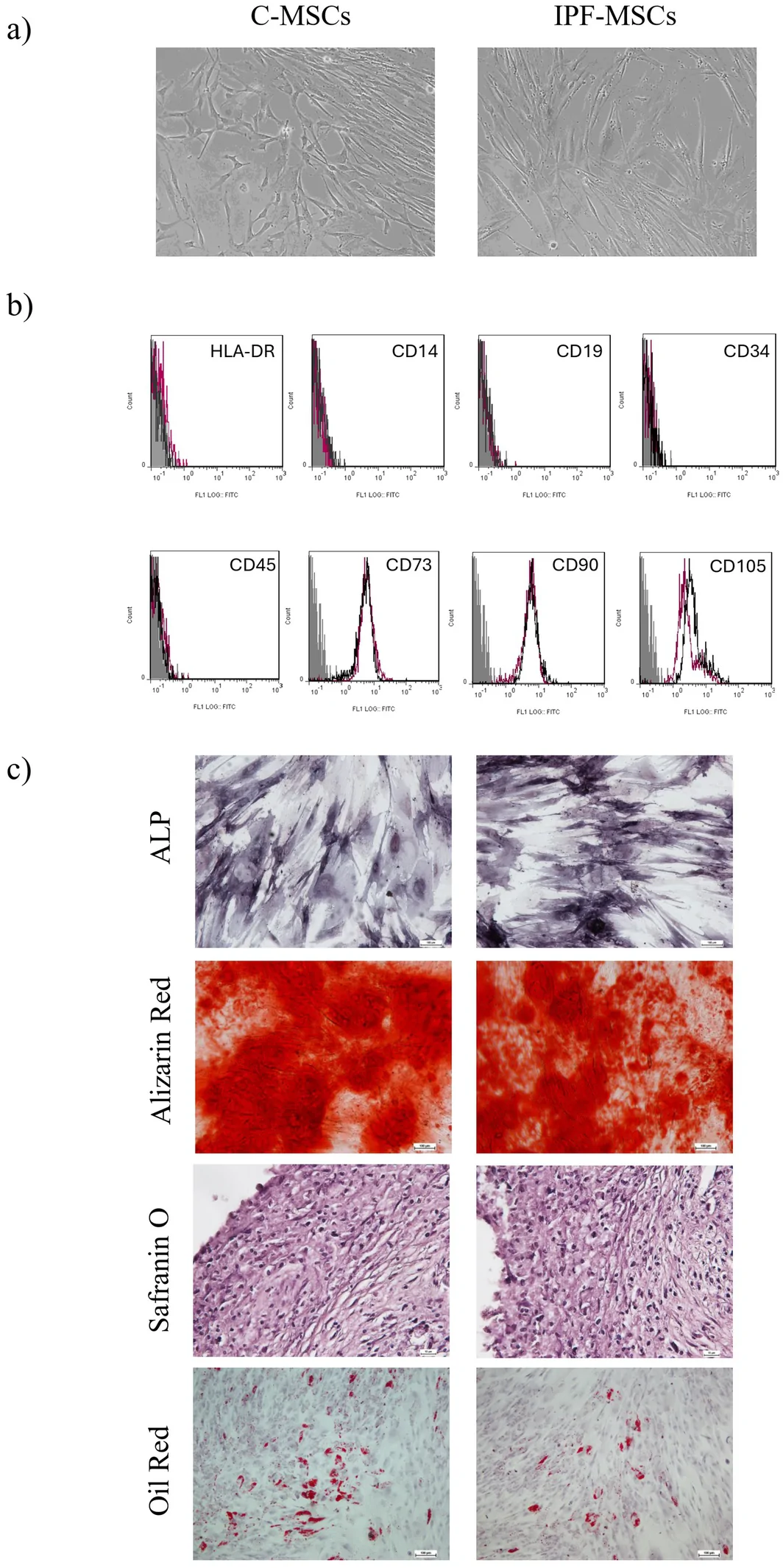
Characterization of mesenchymal stromal cells (MSCs). (a) Representative phase‐contrast microscopy images of mesenchymal stromal cells (MSCs) isolated from control lung tissue (C‐MSCs) and idiopathic pulmonary fibrosis lung tissue (IPF‐MSCs) after 14 days in culture (10× magnification). (b) Representative flow cytometric analysis of MSC surface marker expression. Gray histograms indicate the isotype control (IgG1‐FITC), whereas colored histograms represent representative C‐MSC and IPF‐MSC samples. (c) Multilineage differentiation potential of MSCs. Osteogenic differentiation was evaluated by alkaline phosphatase (ALP) activity at day 7 and Alizarin Red S staining at day 21. Chondrogenic differentiation was assessed by Safranin O staining at day 28, and adipogenic differentiation by Oil Red O staining at day 21. Scale bars: 100 μm (osteogenic differentiation) and 10 μm (chondrogenic and adipogenic differentiation).

Interestingly, IPF‐MSCs exhibited remarkably restricted miRNA dysregulation. Only three miRNAs were significantly downregulated compared with control MSCs: hsa‐miR‐373‐3p (log2FC = −4.01), hsa‐miR‐486‐5p (log2FC = −1.99), and hsa‐miR‐449a (log2FC = −3.86) (Figure 2a,b).

**FIGURE 2:**
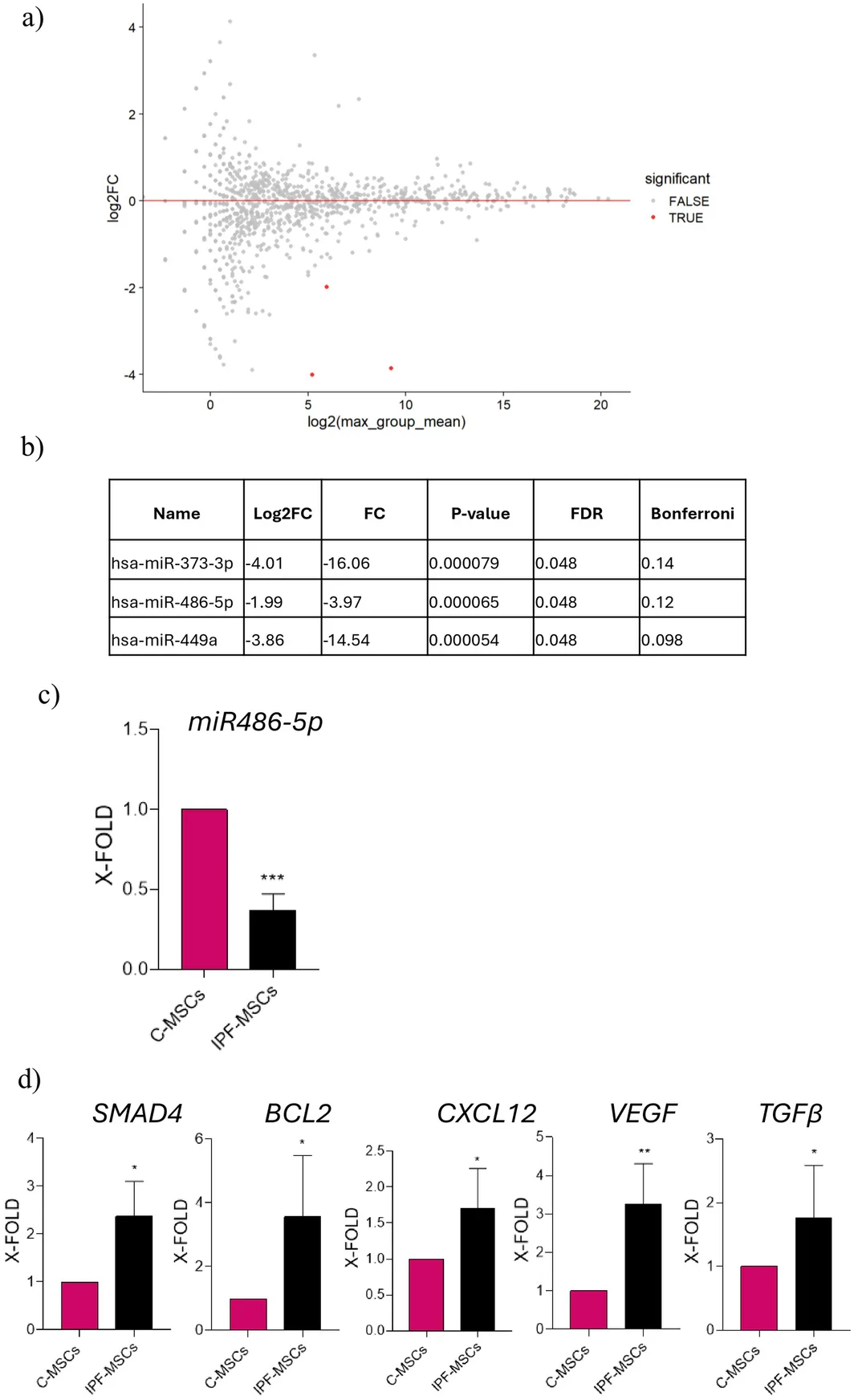
Validation of differentially expressed miRNAs and analysis of selected downstream target genes in IPF‐derived MSCs. (a) Volcano plot showing the selective downregulation of hsa‐miR‐373‐3p, hsa‐miR‐486‐5p, and hsa‐miR‐449a in IPF‐MSCs. (b) Summary of differentially expressed miRNAs identified by small RNA sequencing. Significant differential expression was defined as FDR < 0.05. Bonferroni, Bonferroni corrected *p*‐value; FC, fold‐change; FDR, false discovery rate; log2FC, log2 fold‐change. (c) qRT‐PCR validation of miR‐486‐5p expression in C‐MSCs and IPF‐MSCs. Relative expression was normalized to the endogenous control U6 and expressed as fold change relative to C‐MSCs. (d) qRT‐PCR analysis of selected validated target genes. Expression of the experimentally validated direct targets SMAD4 (miR‐486‐5p) and BCL2 (miR‐449a), together with CXCL12, VEGF, and TGF‐β, genes associated with pathways potentially influenced by miR‐373‐3p dysregulation, was analyzed in C‐MSCs and IPF‐MSCs. Gene expression was normalized to endogenous controls and expressed as fold change relative to C‐MSCs. Data are presented as mean ± SEM. *p* < 0.05 and *p* < 0.01 versus C‐MSCs.

This highly selective pattern suggests that MSC dysfunction in IPF may not require broad miRNA reprogramming, but rather disruption of a limited number of regulatory nodes with disproportionate biological impact.

Among the identified miRNAs, miR‐486‐5p is known to exert antifibrotic activity through inhibition of TGF‐β1/SMAD signaling [3]. Its downregulation is therefore consistent with enhanced fibroblast activation and extracellular matrix deposition. miR‐449a has been implicated in autophagy regulation and cellular stress responses through BCL2 targeting, and reduced expression has previously been associated with fibrotic remodeling [4]. In contrast, miR‐373‐3p has mainly been linked to epithelial–mesenchymal transition and epithelial biology [5], while its functional role in MSCs remains poorly characterized. To validate the biological relevance of these findings, we performed qRT‐PCR validation of miR‐486‐5p (Figure 2c) together with expression analysis of selected validated target genes. Consistent with the sequencing results, qRT‐PCR confirmed the downregulation of miR‐486‐5p, while the direct targets SMAD4 (miR‐486‐5p) [6] and BCL2 (miR‐449a) [7] were significantly upregulated (Figure 2d).

In line with the downregulation of miR‐373‐3p [8], qPCR analysis revealed a concomitant upregulation of TGF‐β, CXCL12, and VEGF (Figure 2c), thereby providing independent biological support for the observed dysregulation of this miRNA and its associated indirect downstream targets in IPF pathogenesis.

The restricted nature of the miRNA signature may be consistent with the marked molecular heterogeneity that characterizes IPF. Rather than exhibiting diffuse regulatory disruption, MSCs may preserve substantial transcriptional stability while selectively losing specific antifibrotic regulatory mechanisms. This interpretation is consistent with the concept of distinct IPF endotypes and may partly explain the heterogeneous clinical behavior observed across patients.

## 1 Limitations

The main limitation of this study is the small sample size, including only five IPF patients and five nonfibrotic controls, which limits the statistical power and generalizability of the findings. In addition, given the limited cohort size and the marked biological heterogeneity of IPF, alternative explanations for the observed differences in miRNA expression, including inter‐individual variability or other disease‐related factors, cannot be excluded. Therefore, these findings should be considered preliminary and hypothesis‐generating.

## 2 Conclusion

Our findings identify a restricted pattern of miRNA dysregulation in IPF‐derived MSCs, characterized by the selective downregulation of miR‐373‐3p, miR‐486‐5p, and miR‐449a. This pattern may reflect the selective impairment of specific regulatory mechanisms rather than widespread miRNA reprogramming in IPF‐MSCs. Larger independent cohorts and dedicated mechanistic studies are required to validate these findings and clarify the contribution of these miRNAs to IPF pathogenesis.

## Author Contributions

M.B. and M.O. contributed equally as senior authors. M.D.V. and P.C. performed experiments and analyzed data. F.M. and F.G. verified data and critically revised the manuscript. M.B. and M.O. conceived the study, interpreted the data, and wrote the manuscript. The authors meet the criteria for authorship as recommended by the International Committee of Medical Journal Editors (ICMJE). All authors reviewed and approved the final manuscript.

## Funding

This independent investigator‐initiated study was supported by a nonconditional grant from Boehringer Ingelheim.

## Ethics Statement

All procedures were conducted in accordance with the Declaration of Helsinki and approved by the Territorial Ethics Committee (CET), Protocol 293/2023.

## Consent

Written informed consent was obtained from all participants.

## Conflicts of Interest

Prof. Bonifazi and Prof. Orciani received a nonconditional grant from Boehringer Ingelheim during the conduct of this study. The sponsor had no role in study design, analysis, interpretation, or manuscript preparation. The remaining authors declare no competing interests.

## Data Availability

Datasets generated during the current study are available from the corresponding authors upon reasonable request.
